# Supplementary material for: Complexome profiling of the Chlamydomonas psb28 mutant reveals TEF5 as an early PSII assembly factor
Source: Plant Cell. 2025 Mar 17;37(6):koaf055. doi: 10.1093/plcell/koaf055 (PMC12164590; doi:10.1093/plcell/koaf055)
Supplement: koaf055_Supplementary_Data [file koaf055_supplementary_data.zip › psb28_interactive.zip!/plots/intensity/qualityPlots/peptides/FAD5A_Cre09.g397250.t1.2.html]

### Cre09.g397250.t1.2

Trivial name: FAD5A  
SeqCoverage [median]: 10.60  
Mass: 46.87 kDa  
  
 

| Sequence | MissedCleavage | Mass [Da] | wtIntensity | muIntensity | Score |
| --- | --- | --- | --- | --- | --- |
| TAQMEEPGVGR | 0 | 1173.54 | 412.34 | 636.74 | 79.97 |
| VAEVVAPTTTLGDAAAAAANDAVR | 0 | 2253.16 | 709.64 | 0.00 | 60.28 |
| VFLSDVATK | 0 | 978.54 | 769.80 | 0.00 | 71.38 |
|  |  |  | 1891.8 | 636.7 |  |
